# How can effective communication help radiographers meet the expectations of patients—COMMUNICATION—a joint statement by the ESR & EFRS

**DOI:** 10.1186/s13244-024-01868-5

**Published:** 2024-12-19

**Authors:** Charlotte Beardmore, Andrew England, Cheryl Cruwys, Dominique Carrié, on behalf of the European Federation of Radiographer Societies,, and the European Society of Radiology

**Affiliations:** 1 European Society of Radiology Patient Advisory Group (ESR–PAG), Vienna, Austria; 2 European Federation of Radiographer Societies (EFRS), Cumieira, Portugal; 3 Society and College of Radiographers, London, UK; 4 University College Cork, Cork, Ireland

**Keywords:** Radiography, Patient/practitioner communication, Patient-centred radiology, ESR/EFRS activities, Radiographers

## Abstract

**Abstract:**

The Patient Advisory Group (PAG) of the European Society of Radiology, in collaboration with the European Federation of Radiographer Societies (EFRS), aims to highlight, in this short paper, the important role that communication plays when trying to meet patients’ expectations throughout their imaging journey in a radiology department. The interactions with radiography professionals carrying out diagnostic or interventional procedures are critical in supporting high-quality patient care and patients’ expectations. The key areas of consideration have been summarised in an easy-to-remember mnemonic: **COMMUNICATION**. There are different healthcare systems and medical imaging services across Europe, and healthcare providers should be mindful, when setting up new operational procedures, of the need for processes and systems to support the delivery of patient-centred care. At times when new or improved technology is being introduced, such as artificial intelligence applications, telemedicine, robotisation of interventional procedures, and digitised records, the impact on patient-radiographer communication and interactions should be considered.

**Critical relevance statement:**

Effective communication helps radiographers meet patients’ expectations by ensuring clear explanations, reducing anxiety, fostering trust, and improving cooperation during procedures. This enhances patient satisfaction, safety, and the overall quality of care, aligning with professional standards and patient-centred healthcare.

**Key Points:**

- Patient-centred imaging services are key to meeting patients’ demands.
- Radiography professionals in radiology departments and medical imaging services should always communicate effectively with patients.
- This ESR-Patient Advisory Group publication attempts to summarise the key areas that should be embedded in patient communication.
- The ‘COMMUNICATION’ statement can be used as a reminder to all radiography professionals to work to improve patient-radiographer interactions and provide patient-focused services.

## Introduction

The European Society of Radiology Patient Advisory Group (ESR-PAG) was created in 2013 [1]. Its goal is to bring together patients, the public, and imaging professionals to positively influence advances in the field of radiology to the benefit of patients in Europe and brings together different European representatives from patient groups, as well as radiologist and radiographer members of the various ESR committees and from the European Federation of Radiographer Societies (EFRS). The ESR-PAG aims to develop and maintain the relationship between healthcare professionals and patients, to improve patients’ knowledge about different imaging modalities and to advocate for a patient-centred approach to the practice of medical radiology in Europe. The ESR-PAG includes a radiographer representative nominated from the EFRS, and the Federation’s role is to represent, promote and develop the profession of radiography in Europe, and therefore this collaboration is important in supporting service improvements for patients. Since 2013, the ESR-PAG has been involved in numerous ESR initiatives, including the International Day of Radiology (IDoR), the ESR-Patient Information website, the EuroSafe Imaging Campaign, contributions to ESR publications, the European School of Radiology (ESOR, Communication course), the ESR-PAG social media team and the Esperanto Patient Satisfaction Questionnaire Audit [2]. Every year, at the European Congress of Radiology in Vienna, the ESR-PAG organises dedicated sessions, which provide a platform for listening to patient representatives and for dialogue with healthcare practitioners, including radiologists and radiographers.

The ESR-PAG works to develop patient-focused initiatives, and this paper follows a publication from the ESR-PAG which focused on ‘What radiologists need to know about patient’s expectations’ [3] and an EFRS publication on the importance of patient engagement and the patient voice within radiographic practice [4]. This paper aims to highlight to radiographers and the medical imaging community the importance of effective communication in delivering patient-centred care.

The principles outlined below may form the basis for standards, which could be developed or incorporated into existing imaging standards, and become the basis for future clinical audits.

## C.O.M.M.U.N.I.C.A.T.I.O.N

### Compassionate care

High-quality compassionate patient care should be at the forefront of radiographic practice [5, 6]. Introduce yourself to every patient using “Hello my name is….and I am the radiographer responsible for your imaging today” [7].

### Organised

Communication with patients or carers should be carefully planned and organised. Provision of information about the imaging examination should be made available in written, verbal and/or other formats to enable all patients to feel prepared for their examination.

### Medical terms

Avoidance of the use of medical jargon is important and therefore all forms of communication should use patient-friendly language, and as required, individualised to the patient [5]. Using pictures with written information to explain complex procedures may be helpful.

### Make a difference

Ask the patient ‘what matters to them’ in order to provide the best care suited to each specific patient.

### Understanding

Checking understanding is paramount in effective patient care. Radiographers should support patients in understanding their imaging procedure and the risk and benefits of the examination. Taking time to listen to the patient, in order to be confident that the patient’s questions are answered, is essential [8].

### Notice

Patients or carers should be provided with information about examinations in advance, in order to understand the purpose of the examination and what will be involved, so that they can prepare.

### Informed

Patients or carers should remain informed of their radiology procedures during and after their care, for example, when and how they will receive their results and any other aftercare requirements. Patients should be provided with a contact telephone number for the departments. It is important that patients, family members or carers have access to easy-to understand information so that they can make informed decisions [9].

### Consent

Informed consent is the cornerstone of effective and safe medical practice and is an essential part of the process following information given to patients or carers.

### Assurance

Radiographers should be able to assure patients or carers that the professionals delivering services have the required education and training to support the safe and effective delivery of the imaging examination.

### Trust

Mutual trust should exist between patients and radiographers, including other healthcare professionals. Confidentiality must be maintained, and information only shared with a patient’s consent or when legally required to do so. Creating safe spaces for confidential discussions is important within departments.

### Interpersonal communication

Should be effective and individualised. For example, adaptations may be required depending on the patient’s age, their language skills, and if the patient has any disabilities [4, 10, 11].

### Openness

Be open and honest with patients and their carers when something has gone wrong with their care. Listen to concerns from patients or carers and communicate that you will follow up promptly, appropriately escalating your concerns where necessary in line with policies and procedures [12].

### Needs

Communication by radiographers should be responsive and address the needs of patients. It is important that, where possible, the needs of the patient are identified prior to the examination. Patients with specific needs should be encouraged to bring along a family member, friend or carer to ensure the most appropriate care and support is provided to patients [13, 14].

## Conclusion

**Fig. 1:**
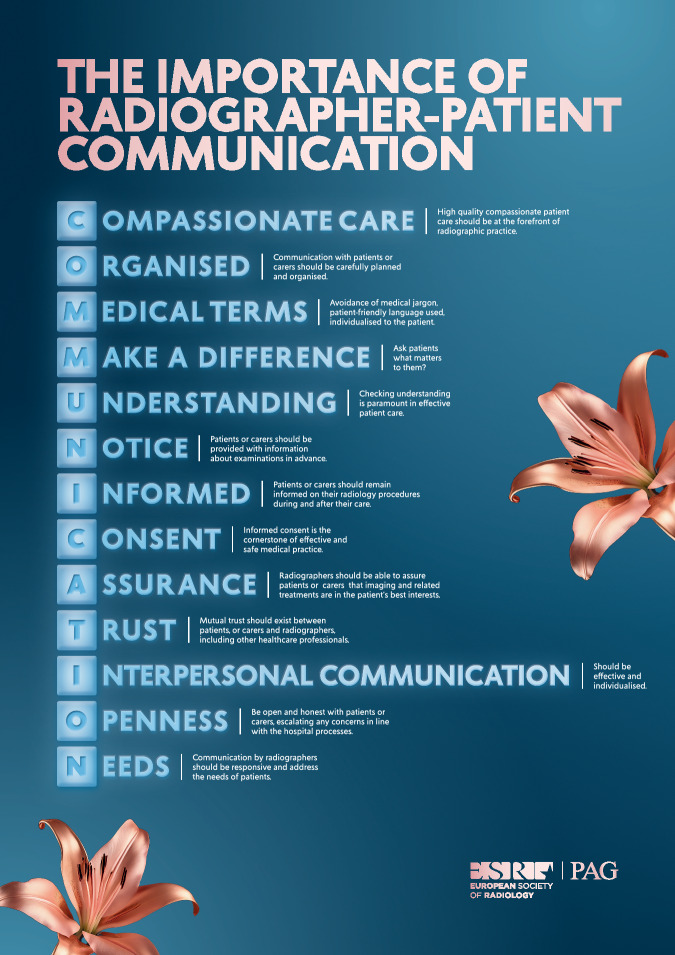
Illustration of the menmonic COMMUNICATION

Radiography teams within Radiology services should always be striving to review their existing processes to ensure that all the processes and protocols support the opportunity for good communication with patients. Incorporating the mnemonic, *COMMUNICATION* (summarised in Fig. 1) serves as a useful prompt, with each point serving as a useful reminder to support excellent daily interactions with patients. The ESR-PAG, together with the EFRS, encourages the use of this guidance to benefit the patient—the most important stakeholder in radiology—with the aim to improve patient-radiographer communication in medical imaging in Europe to ultimately meet patients’ expectations.

## Data Availability

No complex statistical methods were necessary for this paper.

## Abbreviations

EFRS: European Federation of Radiographer Societies
ESR: European Society of Radiology
ESR-PAG: European Society of Radiology Patient Advisory Group
PAG: Patient Advisory Group
